# Desirable Uniformity and Reproducibility of Electron Transport in Single‐Component Organic Solar Cells

**DOI:** 10.1002/advs.202205040

**Published:** 2023-01-19

**Authors:** Haixia Hu, Xinyu Mu, Bin Li, Ruohua Gui, Rui Shi, Tao Chen, Jianqiang Liu, Jianyu Yuan, Jing Ma, Kun Gao, Xiaotao Hao, Hang Yin

**Affiliations:** ^1^ School of Physics State Key Laboratory of Crystal Materials Shandong University Jinan 250100 P. R. China; ^2^ Institute of Functional Nano & Soft Materials (FUNSOM) Jiangsu Key Laboratory for Carbon‐Based Functional Materials and Devices Collaborative Innovation Center of Suzhou Nano Science and Technology Soochow University Suzhou 215123 P. R. China; ^3^ Jiangsu Key Laboratory of Advanced Negative Carbon Technologies Soochow University Suzhou Jiangsu 215123 P. R. China; ^4^ Key Laboratory of Mesoscopic Chemistry of Ministry of Education School of Chemistry and Chemical Engineering Nanjing University Nanjing 210023 P. R. China

**Keywords:** Electron transport, intrachain transport, single‐component organic solar cells, tight‐binding model

## Abstract

Despite the simplified fabrication process and desirable microstructural stability, the limited charge transport properties of block copolymers and double‐cable conjugated polymers hinder the overall performance of single‐component photovoltaic devices. Based on the key distinction in the donor (D)–acceptor (A) bonding patterns between single‐component and bulk heterojunction (BHJ) devices, rationalizing the difference between the transport mechanisms is crucial to understanding the structure–property correlation. Herein, the barrier formed between the D–A covalent bond that hinders electron transport in a series of single‐component photovoltaic devices is investigated. The electron transport in block copolymer‐based devices is strongly dependent on the electric field. However, these devices demonstrate exceptional advantages with respect to the charge transport properties, involving high stability to compositional variations, improved film uniformity, and device reproducibility. This work not only illustrates the specific charge transport behavior in block copolymer‐based devices but also clarifies the enormous commercial viability of large‐area single‐component organic solar cells (SCOSCs).

## Graphical abstract

The electron transport in single‐component organic solar cells based on push‐pull electronic structure experiences potential barriers and wells, hindering the intrachain transport process with a high threshold electric field to drive charge carriers. However, such single‐component cells demonstrate exceptional advantages due to the additional transport channel, involving desirable film uniformity, and reproducibility.

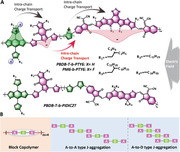

## 1 Introduction

Single‐component organic solar cells (SCOSCs) based on a single photoactive component with covalently linked electron‐donating and electron‐accepting moieties have attracted increasing attention owing to their simplified fabrication process and unique properties (e.g., well‐controlled morphology, excellent miscibility in organic solvents, and exceptional microstructural stability).^[^ ^1^, ^2^, ^3^, ^4^, ^5^, ^6^, ^7^, ^8^, ^9^, ^10^, ^11^, ^12^, ^13^, ^14^, ^15^ ^]^ However, the performances of SCOSCs are inferior to that of their bulk heterojunction (BHJ) counterparts. Fast charge recombination, narrow absorption region, inefficient charge transport process, high energy loss, and uncontrolled intermolecular arrangement ^[^ ^16^, ^17^, ^18^, ^19^, ^20^, ^21^ ^]^ impede the SCOSCs performance. To optimize the device performance, it is essential to achieve the desirable properties of SCOSCs, and thus overcome the limitations. However, the difficulty in obtaining effective morphological control to form highly efficient charge transport pathways and to achieve the desired vertical orientation in films with anisotropic crystals limit the device performance.^[^ ^22^, ^23^ ^]^ Thus, the evaluation of charge carrier transport properties of the SCOSCs is challenging because the information obtained from polycrystalline microstructures, such as the reticular fibre structures, crystallinity, and regioregularity, is much more complicated than that obtained from D–A blend films.

**Figure 1:**
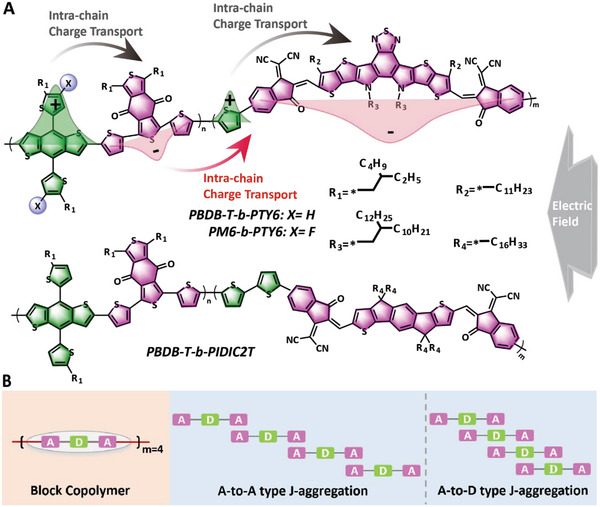
Simplified information about electron transport and modeling. A) schematic diagram of intra‐chain electron transport for block copolymer and corresponding chemical structures, where the green and pink segments separately represent the electron‐push fused ring group and electron‐pull groups. The gray arrow indicates an externally applied electric field. B) Simplified information for three models with four repeat units, namely, the block copolymer, A‐to‐A type J‐aggregation and A‐to‐D type J‐aggregation model.

The single‐component photovoltaic materials based on intramolecular heterojunction are classified into block copolymers with “in‐chain” acceptor groups,^[^ ^24^, ^25^, ^26^, ^27^ ^]^ “double‐cable” conjugated polymers with pendant acceptor groups,^[^ ^6^, ^10^, ^22^, ^28^, ^29^ ^]^ and D–A molecular dyads.^[^ ^4^, ^11^, ^30^, ^31^, ^32^ ^]^ The linker connecting the D and A moieties merges the functions of light absorption, exciton dissociation, and charge transport into one molecule, resulting in distinctly different molecular packing patterns and transport properties of single‐component devices compared to that of the blended devices.^[^ ^33^, ^34^, ^35^, ^36^ ^]^ Conjugated block copolymers are a typical category of single component devices in which there is an alteration of electron‐rich (D/push) and electron‐deficient (A/pull) moieties throughout the polymer backbone.^[^ ^37^, ^38^ ^]^ Delocalized charge carriers on the donor or acceptor block chains can undergo an intrachain transport process within the conjugation length.^[^ ^39^, ^40^, ^41^ ^]^ Block copolymers are expected to provide a continuous charge transport pathway through planar self‐assembly to form ordered nanophase‐separated structures. However, the electron‐push structure of the donor blocks and the electron‐pull structure of the acceptor blocks result in alternating potential barriers and wells at their links, hindering the intrachain electron transport process. Prior reports have experimentally demonstrated that block copolymers weaken *π*‐stacking, implying the short‐range order of long copolymer chains, which may be attributed to the increased irregularity of polymer backbone and defect states.^[^ ^1^, ^2^ ^]^ **Figure** **1**A depicts the schematics of the intrachain electron transport model in single‐component photovoltaic devices. In conventional state‐of‐the‐art BHJ films, the *π*–*π* interaction is a major packing pattern that facilitates electron transport.^[^ ^42^, ^43^, ^44^ ^]^ The existence of traps not only reduces the current density and performance of the SCOSCs by trapping free carriers, but also alters the electric field dependence.^[^ ^45^ ^]^ The trap states originating from structural defects in one or more homopolymers impurities, tri‐block copolymers, irregular long chains, and geminate pairs in SCOSCs significantly influence the charge carrier transport properties.^[^ ^46^, ^47^, ^48^ ^]^ However, a clear correlation between the transport properties and device performance has not been fully described.

Herein, we systematically studied the charge carrier transport behaviors in SCOSCs employing experimental and theoretical methods. Three conjugated block copolymer‐based single‐component devices, namely PBDB‐T‐*b*‐PIDIC2T, PM6‐*b*‐PTY6, and PBDB‐T‐*b*‐PTY6, were selected as model systems and compared with BHJs. Quantitative capacitance spectroscopy measurements indicated that the conjugated block copolymer‐based devices possessed higher carrier trap concentrations than the BHJ devices. To examine the carrier mobility in single‐component devices, we employed the trap‐limited space‐charge‐limited current (trap‐SCLC) technique and admittance spectroscopy (AS). The results demonstrate that the SCOSCs exhibit lower charge carrier mobility values and the electron transport is highly dependent on the electric field. The block copolymer maintains a relatively stable electron mobility, which might be due to the formation of percolation transport networks when inert polystyrene (PS) was added as an extrinsic component. Theoretically, by employing a tight‐binding model combined with a nonadiabatic evolution method, we compared the threshold electric field strengths for the electron transport and the transport velocities in the block copolymer‐based models. A‐to‐A type J‐aggregation and A‐to‐D type J‐aggregation models were used to observe a higher threshold electric field strength and a lower transport velocity for block copolymer‐based models. Remarkably, the stable conjugated long‐chain structures of the block copolymer constructed additional intrachain electron transport channels, leading to favorable uniformity and reproducibility in charge transport and device performance of SCOSCs. This work reconsiders the origins of the inferior transport properties and confirms the positive impacts of the potential applications of single‐component devices.

## 2 Results and Discussion

### 2.1 Characteristics of Electron and Hole Transport Properties

**Figure 2:**
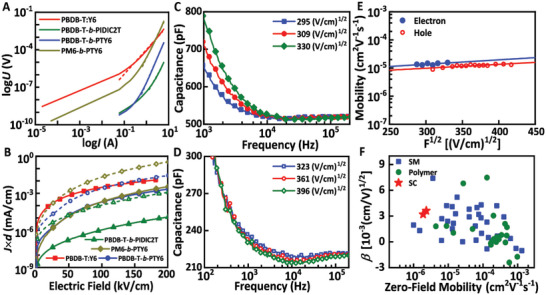
Basic transport properties characteristics of the target systems. A) Schematic illustration of the log *I*‐log *U* characteristics. B) The dependence of the *J* × *d* value with the applied electric field exhibited by three single‐component systems (i.e., PBDB‐T‐*b*‐PIDIC2T, PBDB‐T‐*b*‐PTY6, and PM6‐*b*‐PTY6) and PBDB‐T:Y6 BHJ films, where the solid line is based on the SCLC model and the dashed line is based on the trap‐SCLC model. Bias voltage dependent capacitance‐frequency spectra of C) electron‐only device and D) hole‐only device. E) Field‐dependent carrier mobilities for PBDB‐T‐*b*‐PTY6 based high‐efficiency SCOSCs. F) A reference summary of small molecular (SM)‐based and polymer‐based blending films and PBDB‐T‐*b*‐PTY6 films in this work on Poole–Fenkel slope as a function of zero‐field mobility.

We fabricated electron‐only and hole‐only devices and investigated the charge transport mechanism in single‐component polymer and BHJ‐type films. Three block copolymer materials, namely, PBDB‐T‐*b*‐PIDIC2T, PM6‐*b*‐PTY6, and PBDB‐T‐*b*‐PTY6, were selected as model systems. The PBDB‐T:Y6 BHJ device corresponding to the PBDB‐T‐*b*‐PTY6 device with the highest performance efficiency 9.85% was adopted as a control in this work. The SCLC and AS measurements were performed to evaluate the charge carrier transport behavior in such films. The detailed device fabrication process and experimental mechanism have been well documented in the Experimental Section and the Supporting Information sections. **Figure** **2**A shows the characteristic current–voltage curves, which shift from the ideal square law (*I*∝*U* ^2^) in BHJ blending films to voltage raised to the fourth power (*I*∝*U* ^4^) in all three single‐component devices. This deviation can be attributed to the presence of excessive traps.^[^ ^49^, ^50^, ^51^ ^]^ As shown in Figure 2B, such trap states also reduce the current density, resulting in a decrease in electron mobility, and hence in the device performance. To investigate the charge transport properties of the different single‐component films accurately, the modified SCLC model with trap‐SCLC was used to extract carrier mobilities, which can be expressed using Equation (1)

(1) $$J_{\mathrm{SCL}}\times d\;=\frac{9}{8}\;\varepsilon_{0}\varepsilon_{r}\mu_{0}\theta \exp\left(0.89\beta \sqrt{F}\right)F^{2}$$

where *J*_SCL_ is the space‐charged‐limited current density, *d* is the thickness of the tested active layer, *ε*_0_ is the permittivity of vacuum, *ε*_r_ is the relative permittivity, *θ* is defined as the trap factor correlated with free and trapped carrier densities, *µ*_0_ is the zero‐field carrier mobility, *β* is the Poole–Frenkel slope, and *F* is the applied electric field.^[^ ^45^, ^52^, ^53^ ^]^ The value of *θ* can be determined by frequency‐dependent capacitance spectra under different bias voltages, as shown in Figure S1 (Supporting Information). All extracted trap densities of the single‐component devices were much higher than that of the BHJ device, as seen in the C^−2^ versus V plot (Figure S2, Supporting Information). The value of *θ* for these three single‐component devices were all around 10^−2^, and PM6‐*b*‐PTY6 with the highest trap concentration of 4.2 × 10^−23^ m^−3^ showed a lowest *θ* value of 0.01. For PBDB‐T‐*b*‐PTY6, the *µ*_0_ of 8.7 × 10^−5^ cm^2^ V^−1^ s^−1^ was obtained from the trap‐SCLC fitting, which is much lower than 6 × 10^−4^ cm^2^ V^−1^ s^−1^ of the PBDB‐T:Y6 BHJ film. The electron energetic disorder (*σ*_e_) was determined from the Gaussian disorder model fitting for electron mobilities at different temperatures and reflects the trap states inside the devices.^[^ ^54^, ^55^ ^]^ As shown in Table S1 (Supporting Information), compared to *σ*_e_ of 54 meV for the PBDB‐T:Y6 BHJ film, the single‐component‐based PBDB‐T‐*b*‐PTY6, PBDB‐T‐*b*‐PIDIC2T, and PM6‐*b*‐PTY6 films exhibit higher *σ*_e_ values of 60, 62, and 72 meV, respectively.

The topographical images (Figure S3, Supporting Information) of the BHJ and block polymer films obtained using atomic force microscopy show less textured surface structures of the PBDB‐T‐*b*‐PTY6, PBDB‐T‐*b*‐P2TIDIC, and PM6‐*b*‐PTY6 devices, leading to smaller values of root‐mean‐square (RMS) surface roughness. The unfavorable nanofiber morphology of films in block copolymers may result in decreased carrier mobility; however, the internal charge carrier transport mechanism remains unclear. To address this issue, we performed AS to investigate the field‐dependent properties of single‐component‐based films. The field dependent electron mobilities *µ*_e_ of the organic semiconductors can be calculated using the following Equation (2)

(2) $$\mu_{e}\;\left(F\right)=\mu_{0,e}\;\exp\left(\beta_{e}\sqrt{F}\right)$$

where the notations are the same as defined before.^[^ ^56^ ^]^ The PBDB‐T‐*b*‐PTY6 film shows relatively lower field‐dependent electron and hole mobilities of ≈10^−5^ cm^2^ V^−1^ s^−1^. The Poole–Frenkel effect describes the electric field dependence of charge carrier transport properties and is associated with the required electric field for electrons in the localized state to be pulled in organic semiconductors.^[^ ^57^, ^58^ ^]^ Figure 2F shows a plot of zero‐field mobility versus Poole–Fenkel slope for the small molecular (SM)‐based and polymer‐based blending films and the single‐component PBDB‐T‐*b*‐PTY6 films. For most BHJ blending devices, the values of *µ*_0,e_ are in the 10^−5^–10^−3^ cm^2^ V^−1^ s^−1^ range and the *β* values are in the 0.5–4.5 × 10^−3^ (cm V^−1^)^1/2^ range. However, single‐component devices exhibit both low *µ*_0,e_ of 10^−6^ cm^2^ V^−1^ s^−1^ and strong electric field dependence with the value of *β* ≈3 × 10^−3^ (cm V^−1^)^1/2^, indicating inferior charge transport properties in SCOSCs than their BHJ counterparts.

### 2.2 Impact of Inert Polystyrene Molecules on Electron Transport

**Figure 3:**
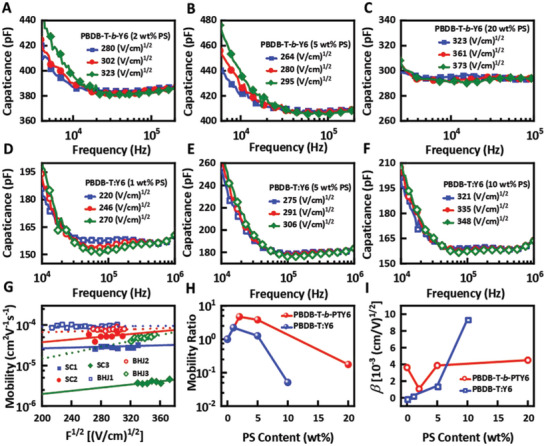
Admittance spectrum and related parameters with different PS contents. The frequency‐dependent capacitance characteristics under different bias voltage of A–C) PBDB‐T‐*b*‐PTY6 devices and D–F) PBDB‐T:Y6 devices with various polystyrene (PS) contents. G) The field‐dependent electron mobilities, H) mobility change ratio, and I) electric field dependent parameters *β* of single‐component material PBDB‐T‐*b*‐PTY6 and the counterpart PBDB‐T:Y6 BHJs with different PS contents.

To distinguish between the charge transport properties of single‐component and BHJ films, inert polystyrene (PS) was added into the single‐component PBDB‐T‐*b*‐PTY6 and PBDB‐T:Y6 BHJ films. **Figure** **3**A–F depicts the frequency‐dependent admittance spectra under different bias voltages in electron‐only devices with variousPS contents. Figure 3G–I summarizes the extracted field‐dependent electron mobility from AS signal, the mobility change ratio and *β* in single‐component and BHJ films under different PS contents. In prior reports, the excellent device performances and morphological stabilities of the SCOSCs under ambient and thermal stress conditions indicate that the block copolymer‐based devices may be insensitive because of the presence of extrinsic substances.^[^ ^5^, ^24^, ^33^ ^]^ The single‐component device exhibits a smoother change in carrier mobility and *β* values on adding PS. PS exhibits a unique functional structure and is used in an appropriate concentration to optimize the performance of OSCs by enhancing electron mobility.^[^ ^59^ ^]^ For both the PBDB‐T‐*b*‐PTY6 and PBDB‐T:Y6 devices with less than 2 wt% PS, an effective increase in electron mobility was observed. The mobility enhancement is more prominent for the PBDB‐T‐*b*‐PTY6 device owing to its miscibility with insulating polymers, forming percolation transport networks.^[^ ^60^, ^61^ ^]^ The impurity molecules, such as insulating materials, may occupy charge carrier transport sites and hinder the carrier transport process.^[^ ^62^ ^]^ For example, when 20 wt% PS was added to the PBDB‐T‐*b*‐PTY6 film, it showed a significantly lower electron mobility of 8.1 × 10^−7^ cm^2^ V^−1^ s^−1^ than electron mobility of 4.6 × 10^−6^ cm^2^ V^−1^ s^−1^ for the intrinsic device. Conversely, in BHJ, the mobility value decreases from intrinsic system from 4.1 × 10^−5^ cm^2^ V^−1^ s^−1^ for the intrinsic device to 2.1 × 10^−6^ cm^2^ V^−1^ s^−1^ for the BHJ films with 10 wt% PS. Regardless of the PS content, the PBDB‐T:Y6 BHJ device maintained a higher electron mobility than the PBDB‐T‐*b*‐PTY6 device. The *β* of pure PBDB‐T:Y6 blending device was −1 × 10^−4^(cm V^−1^)^1/2^, and even with 1 wt% PS, the *β* increased rapidly to 2.5 × 10^−4^ (cm V^−1^)^1/2^ and persisted with increasing PS contents. Starting with pristine PBDB‐T‐*b*‐PTY6, adding a low concentration of PS (<5 wt%) increased the the electron mobility to 2.2 × 10^−5^ cm^2^ V^−1^ s^−1^ and lowered the *β* to 1.0 × 10^−3^ (cm V^−1^)^1/2^ Although introducing a high concentration of PS can increase the *β* values of the PBDB‐T‐*b*‐PTY6 device, the range of the variation is much lower than that of the BHJ devices, implying the favorable stability of the electron transport in single‐component devices Besides the electron transport, we also constructed hole‐only devices as shown in Figure S4 (Supporting Information). The addition of PS reduced the mobility ratio of the hole transport than the electron transport. Unlike the electron transport, neither the PBDB‐T‐*b*‐PTY6 nor PBDB‐T:Y6 hole‐only devices with less than 2 wt% PS exhibited an increase in hole mobility, and a similar trend of lowering was observed. Remarkably, the hole mobility value of 2.9 × 10^−5^ cm^2^ V^−1^ s^−1^ for PBDB‐T‐*b*‐PTY6 devices with 5 wt% PS significantly improved compared to that of 5.9 × 10^−5^ cm^2^ V^−1^ s^−1^ for devices with 2 wt% PS. Even with 1 wt% PS, the hole mobility values of the PBDB‐T:Y6 device reduced rapidly and persisted with increasing PS concentration. Thus, there is a negligible difference in the performance of PBDB‐T‐*b*‐PTY6 devices with different PS concentrations. Therefore, it implies that single‐component materials are highly tolerant to compositional variations and excellent stability of the hole transport behavior.

### 2.3 Tight‐Binding Model Simulation of Carrier Transport Behavior

**Figure 4:**
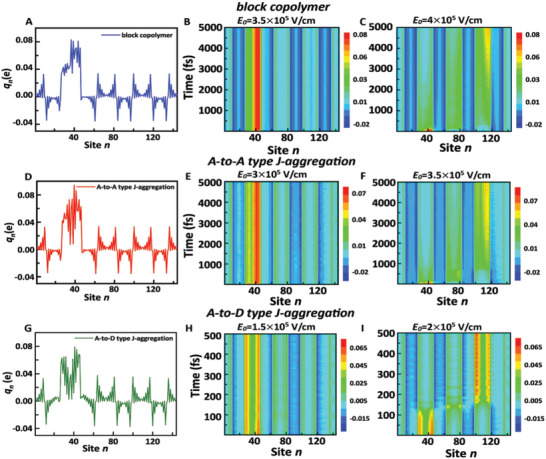
Initial distribution and time dependent dynamic evolution of net charge. A), D), and G) separately presents the initial charge density on each site of the block copolymer, A‐to‐A type J‐aggregation and A‐to‐D type J‐aggregation structural models; time evolutions of the net charges on each site of the corresponding molecular models with different electric fields applied, where B,C) show the result of block copolymer, E,F) A‐to‐A type J‐aggregation, and H,I) A‐to‐D type J‐aggregation.

Single‐component materials also share some common features of BHJ blending films, such as conjugated skeletons and push–pull electronic structures. Figure 1B shows the construction of a simplified structural model based on the information on block copolymers and models of two aggregated morphologies extensively adopted in BHJ films, namely A‐to‐A‐type J‐aggregation and A‐to‐D‐type J‐aggregation models. To accurately explain the charge transport behaviors and their electric field dependences in single‐component materials, we employed an extended Su–Schrieffer–Heeger (SSH) tight‐binding model,^[^ ^63^, ^64^, ^65^ ^]^ which highlights the strong electron‐lattice (*e‐l*) interactions in organic semiconductor materials. Detailed modeling information has been described in the Supporting Information. In a block copolymer, the transition integral of the covalent bond linking fragments is chosen as *t*_b_ = 1.5 eV. Herein, we assume that a negative polaron (i.e., a spatially localized electron charge) is initially generated at the left side of different molecular systems with four polymerized units (*m* = 4). The charge density curves of each grid point are show in **Figure** **4**A–C,D–F,G–I, respectively.

The initial net charge distribution *q_n_* = e(*ρ_n,n_*‐1) of different structural models indicates that there is an intramolecular charge transfer character, caused by the on‐site energy difference between the D and the A units (the details of on‐site energy have been described in the Supporting Information). In addition, it implies that the D units will act as a potential barrier to impede electron transport. As demonstrated in Figure 4B,C, the negative polaron in the block copolymer chain can be transported from the initial position to the right end group, at least under the electric field strength of *E*_0_ = 4.0 × 10^5^ V cm^−1^. However, for the BHJ blending devices (Figure 4E–I), a lower applied external electric field compared to the single‐component devices ensures the efficient transport of negative polarons in organic devices, especially for the A‐to‐D type J‐aggregation‐based devices. The threshold electric field strengths of the devices based on A‐to‐A type J‐aggregation and A‐to‐D type J‐aggregation models were 3.5 × 10^5^ and 2 × 10^5^ V cm^−1^, respectively. The order of the threshold electric field of negative polaron transport is as follows: 4 × 10^5^ V cm^−1^ (block copolymer‐based model) > 3.5 × 10^5^ V cm^−1^ (A‐to‐A type J‐aggregation‐based model) > 2 × 10^5^ V cm^−1^ (A‐to‐D type J‐aggregation‐based model). To obtain the transport velocities of the negative polaron under these three electrical environments, we applied an electric field of *E*_0_ = 4 × 10^5^ V cm^−1^. During the transport process, the evolution of the charge center position of the negative polaron with time is shown in Figure S5 (Supporting Information). The results indicate that the negative polaron has the slowest transport velocity in the block copolymer‐based model, corresponding to its high threshold electric field. The transport velocities of the negative polaron are analogous in the two models of BHJ devices, and the A‐to‐D type J‐aggregation‐based model takes precedence over the A‐to‐A type J‐aggregation‐based model.

### 2.4 Uniformity and Reproducibility Analysis

**Figure 5:**
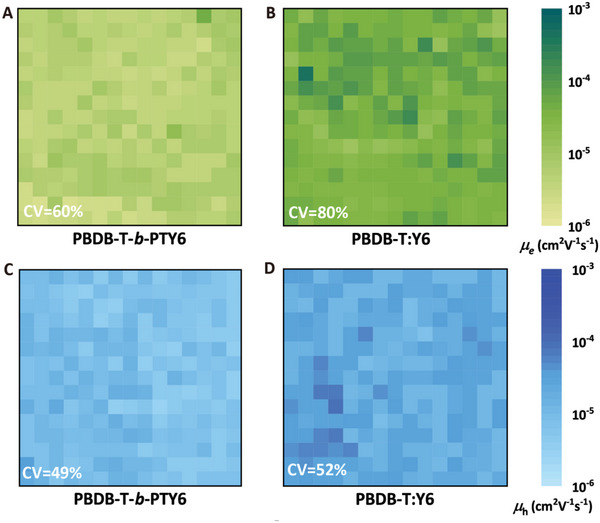
The carrier mobilities site maps. A,B) The electron mobility mapping of PBDB‐T‐*b*‐PTY6 and PBDB‐T:Y6 films, respectively. C,D) The hole mobility mapping of PBDB‐T‐*b*‐PTY6 and PBDB‐T:Y6 films, respectively.

Based on the additional intrachain transport channel of electrons in single‐component materials with irregular long chains, we attempted to exploit other potential advantages in addition to the stability, which has been well discussed.^[^ ^5^, ^29^, ^30^ ^]^ The reproducibility of the performance for different batches of devices and the variability of their performance among various sites in large‐area devices must be considered.^[^ ^66^ ^]^ We measure 15 × 15 individual devices on a substrate with an effective area of 1 cm^2^ using the SCLC technique to evaluate the carrier mobility of each device and uniformity of the charge carrier transport. The dispersion of the carrier mobility at different sites was evaluated using the coefficient of variation (*c_V_*), which can be calculated using Equation (3)

(3) $$c_{v}=\frac{\sigma }{\bar{\mu }}\;$$

where *σ* is the standard deviation of the carrier mobility, and $\bar{\mu }$ is the mean carrier mobility.^[^ ^67^ ^]^ The lower *c_V_* values indicate a lower dispersion of carrier mobilities and higher uniformity of the charge carrier transport. In **Figure** **5**A,B, the PBDB‐T‐*b*‐PTY6 devices as a whole exhibit lower electron mobility with a mean mobility of 5.3 × 10^−6^ cm^2^ V^−1^ s^−1^, whereas the corresponding PBDB‐T:Y6 BHJ devices show a mean mobility of 4.7 × 10^−5^ cm^2^ V^−1^ s^−1^. For PBDB‐T:Y6, the overall mean electron mobility measured using the SCLC method is significantly poor due to the higher *c_V_* value of 80%, whereas other devices show a mean electron mobility of the order of 10^−4^. The high dispersion of the electron mobility of the BHJ‐based large‐area devices implies the inhomogeneity of charge transport, which may be a reason for its decreased efficiency. Remarkably, the PBDB‐T‐*b*‐PTY6 device exhibited superior uniformity of electron mobility with a low *c_V_* value of about 60%. In Figure S11 (Supporting Information), the graph of the ratio of carrier mobility values to mean mobility values versus different devices is shown to visualize the homogeneity. It can be observed that the distribution of carrier mobility values in the vicinity of the mean value is symmetric and narrow for single‐component devices compared to BHJ devices, especially for electrons. The uniformity of the electron mobility is consistent with the uniformity and reproducibility of the different device performances, which are shown in the photovoltaic characteristic (Figures S6 and S7, Supporting Information) and 2D time‐resolved fluorescence imaging (Figure S8, Supporting Information) of films. Moreover, the results of grazing incidence wide‐angle X‐ray scattering (Figure S9, Supporting Information) measurements indicate that single‐component devices have weakened *π*–*π* stacking for electron transport. The high uniformity of the single‐component may be due to the additional intrachain transport channels of the conjugated polymer long chains, increasing the possibility of lateral charge carrier transport. Furthermore, we constructed hole‐only devices, as shown in Figure 5C,D. The similar *c_V_* values of 49% for PBDB‐T‐*b*‐PTY6 and 52% for PBDB‐T:Y6 hole‐only devices confirm that intrachain transport provided by the conjugated long chains enhances the uniformity of charge carrier transport. Thus, it provides a new research perspective to optimize the performance of large‐area devices.

## 3 Conclusion

In summary, we systematically investigated the carrier transport properties based on the unique structure of block copolymers in single‐component devices and revealed their attractive potential for commercial applications. The photovoltaic performance of single‐component devices is still not comparable to that of BHJs, primarily because of the numerous trap states and strong electric field dependence of carriers in single‐component materials revealed by the SCLC and AS measurements. The tight‐binding model calculations demonstrated that because of the push–pull electronic structure the electron transport experienced alternating potential barriers and wells, which hinder the intrachain transport of the charge carrier. This hindered transport results in a single‐component device with a higher threshold electric field to drive charge transport and lower charge transport velocity than the BHJ devices. However, the single‐component material also displays a distinctive advantage of having an additional electron transport channel due to its conjugated long chain. For example, the block copolymer materials maintained a relatively stable electron mobility under the presence of PS as an impurity and excellent uniformity and reproducibility of charge carrier transport and photovoltaic properties on a large scale. This work elucidates the potential barrier effects in single‐component materials with block copolymer structures, which are distinct from that in the BHJ‐based devices in single‐component materials, and this observation significantly contributes to the development of stable and efficient SCOSCs for commercial applications.

## 4 Experimental Section

### Device Fabrication

The electron‐only devices were fabricated with a conventional configuration of ITO/Al (50 nm)/active layer /PDIN/Al. The patterned indium tin oxide (ITO)‐coated substrates were sequentially cleaned in an ultrasonic bath by using detergent, deionized water, acetone, absolute ethyl alcohol, and isopropyl alcohol for 20 min in each step. These glass substrates were then treated by UV‐ozone for 15 min to improve their work function. The 50 nm Al layer deposited onto these cleaned substrates by thermal evaporation is used to block hole carriers. Afterward, these prepared substrates were transferred into a nitrogen‐filled glove‐box. The blending solution with total 24 mg mL^−1^ can be obtained by dissolving an organic donor (PBDB‐T) and acceptor materials (Y6) through 1 vol% CN chloroform (CF) solution. Single‐component solution including PBDB‐T‐*b*‐PIDIC2T (12 mg mL^−1^) or PBDB‐T‐*b*‐PTY6 (15 mg mL^−1^) or PM6‐*b*‐PTY6 (15 mg mL^−1^) in 1 vol% CN chloroform (CF) solution can be obtained. Additive polystyrene (PS) was dissolved in CF with a concentration of 7 mg mL^−1^, when add PS solution into BHJ or single‐component solution with the weight ratio of (0–20 wt%). The bulk heterojunction (BHJ) and single‐component active layer films above 200 nm were fabricated by spin‐coating the blend solution with 2000 rpm for 50 s on the top of Al and then annealed at 120 °C for 10 min in nitrogen glove box. After that, a thin layer of PDIN was spin coated on BHJ layer to contribute to the electron transport at 5000 rpm for 30 s. Finally, the top Al electrodes of 130 nm were deposited on the top of PDIN layer by thermal evaporation to finish the preparation of the whole devices, thus yielding the active area of 0.012 cm^2^ for normal devices through a shadow mask.

### Carrier Mobility Measurements

The current density–voltage (*J*–*V*) characteristic curves of all devices were recorded in a low vacuum environment by employing a computer‐controlled Keithley 2612B. And the charge carrier mobilities of were measured by employing the SCLC method and then calculated by fitting the dark current with the Mott–Gurney square law.

## Conflict of Interest

The authors declare no conflict of interest.

## Supporting information

Supporting Information

## Data Availability

The data that support the findings of this study are available in the supplementary material of this article.
