# Supplementary material for: Desirable Uniformity and Reproducibility of Electron Transport in Single‐Component Organic Solar Cells
Source: Adv Sci (Weinh). 2023 Jan 19;10(8):2205040. doi: 10.1002/advs.202205040 (PMC10015880; doi:10.1002/advs.202205040)
Supplement: Supplementary file 1 — Supporting Information [file ADVS-10-2205040-s001.pdf]

Supporting Information

Desirable Uniformity and Reproducibility of Electron Transport in Single-Component Organic Solar Cells

*Haixia Hu, Xinyu Mu, Bin Li, Ruohua Gui, Rui Shi, Tao Chen, Jianqiang Liu, Jianyu Yuan, * Jing Ma, Kun Gao, * Xiaotao Hao, * and Hang Yin**

This PDF file includes:

Supplementary Text
Figs. S1 to S11
Tables S1 to S6
References (1 to 4) (if applicable—these should refer only to references in the SM)

Supplementary Text

Extended technical descriptions of Materials, the Synthesis of block copolymers, Device fabrications, Admittance Spectroscopy (AS), instruments and characterizations, calculation of trap density analysis, charge carrier density, and full details of device fabrications for hole-only devices and organic solar cells and the tight-binding model and nonadiabatic evolution method.

Materials

The polymer donor PBDB-T, small molecule acceptor Y6 and electron transportation layer material PDIN used in this work were purchased from Solarmer Materials Inc. PEDOT:PSS (Clevios PVP Al 4083) was purchased from Xi'an Polymer Light Technology Corp. The insulating polymer polystyrene (PS) and 1-chloronaphthalene (CN) were supplied by Sigma Aldrich. All materials were used without further purification.

The Synthesis of block copolymers

The synthesis of PBDB-T-b-PIDIC2T and PBDB-T-b-PTY6: Under an inert atmosphere, M1 (36.75 mg, 4.06 mmol, 1 eq.), M2 (31.15 mg, 4.06 mmol, 1 eq.) and Pd(PPh3)4 (3.5 mg) were first dissolved in anhydrous toluene/DMF (2.5/0.25 mL) in a 50 mL reaction flask. The temperature was gradually increased to 110 °C while stirring the reaction mixture to prepare the polymers PBDB-T. After 1 hour, the mixed toluene/DMF solution (2/0.2 mL) of IDIC-Br and Y6-Br (80.19 mg, 4.06 mmol, 1 eq.) and 2,5-bis(trimethylstannyl)thiophene (16.64 mg, 4.06 mmol, 1 eq.) was injected into the reaction flask where PBDB-T was prepared. The reaction mixture was kept under stirring at 110 °C for 24 h. The resulting solution was cooled and precipitated in acetone (100 mL). The residual oligomers and catalyst were removed through Soxhlet extraction with acetone (12 h) and hexane (12 h), and the final block polymer product was extracted using chloroform. The chloroform fraction was then precipitated in acetone (100 mL), and the precipitated products were dried under vacuum at 80 °C for 12 h to obtain PBDB-T-b-PIDIC2T as a dark solid GPC: Mn = 32.6 kg mol−1; PDI = 3.01 and PBDB-T-b-PTY6 as a dark solid (102 mg, 81.6%) GPC: Mn = 24.5 kg mol−1; PDI = 2.78. [1]

The synthesis of PM6-b-PTY6:In an inert atmosphere, M1 (34.8mg, 0.0372 mmol, 1 eq.), M2 (28.5mg, 0.0372 mmol, 1 eq.) and Pd(PPh3)4 (3.5 mg) were first dissolved in anhydrous toluene/DMF (2.5 mL /0.25 mL) in a 50 mL reaction flask. The temperature was gradually increased to 110 °C while stirring the reaction mixture to prepare the donor polymer PM6.

After 1 h, the mixed toluene/DMF solution (2 mL /0.2 mL) of Y6-Br (73.5mg, 0.0372 mmol, 1 eq.) was injected into the reaction flask where the PM6 was prepared. The reaction mixture was kept under stirring at 110 °C for 24 h, and the resulting solution was cooled and precipitated in methanol (100 mL). The residual oligomers and catalyst were removed through Soxhlet extraction with acetone (12 h) and hexane (12 h), and the final block polymer product was extracted using chloroform. The chloroform fraction was then precipitated into methanol (100 mL), and the precipitated products were dried under vacuum at 80 °C for 12 h to obtain the PM6-b-PTY6 as dark solid (92 mg, 82%), GPC: Mn =31.5 kg/mol, PDI = 2.98.[2]

Device fabrication and Measurements:

Hole-only devices: The hole-only devices were fabricated with a conventional configuration of ITO/PEDOT:PSS/ active layer /Spiro-TPD/Au. The PEDOT:PSS was spin-coated onto these cleaned substrates at 4000 rpm for 50 s and annealed at 150 °C for 10 min in ambient atmosphere. Afterwards, these prepared substrates were transferred into a nitrogen-filled glove box. The active layers (>200 nm) were fabricated by spin-coating the blend solution on the top of PEDOT:PSS layer and then annealed. After a 10 nm Spiro-TPD layer deposited by thermal evaporation, and 80 nm Au layer was deposited on the substrate. The current density-voltage (J-V) characteristic curves of all devices were recorded In a low vacuum environment by employing a computer-controlled Keithley 2612B.

Organic solar cells (OSCs):

The inverted structure is ITO/ZnO/active layer/MoO_3/Ag and the conventional structure is ITO/PEDOT:PSS/active layer/PDIN/Al. For the device fabrication, the ITO substrates were ultrasonic cleaned in acetone, water, acetone, and isopropyl alcohol. First, for the inverted device, sol–gel ZnO was spin-coated onto the pre-cleaned ITO substrate at 4000 rpm for 40 s and then the substrates were baked at 200 °C for 10 min under ambient condition; for the conventional one, the PEDOT:PSS spin-coated onto the pre-cleaned ITO substrate at 4000 rpm for 50 s and then the substrates were baked at 150 °C for 10 min under ambient condition. Subsequently, PBDB-T:Y6 (D:A ratio 1:1.2, total 16 mg/mL in chloroform) or PBDB-T-*b*-PIDIC2T (8 mg/ml in chloroform) or PBDB-T-*b*-PTY6 (10 mg/ml in chloroform) or PM6-*b*-PTY6 (10 mg/ml in chloroform) solutions were deposited onto the ZnO or PEDOT:PSS layer for 40 s, at spin rate of 3000 rpm in nitrogen glove box. After thermal annealing at 120 °C for 10 min in the glove box, finally, for the inverted device, MoO_3 at a speed of 0.2 Å/s (8 nm), and Ag at a speed of 2 Å/s (100 nm) layers were thermally evaporated at a pressure of 5 ×

10^{-6} mbar to accomplish the device fabrication; for the conventional device, PDIN (2 mg/mL in methanol) was spin-coated onto the active layer at 5000 rpm for 30 s and Al at a speed of 2 Å/s (100 nm). The J–V characterization was performed by Keithley 2400 digital source meter under simulated AM 1.5G solar irradiation at 100 mW cm^{-2} . The device area was 0.0725 cm^2 , solar cell devices were measured in forward scan (−1.0V → 1.0V, step 0.0125V, scan rate: 0.1V s^{-1}) without an illumination mask in glovebox.

Admittance Spectroscopy (AS)

Admittance Spectroscopy (AS) can be used to evaluate the charge carrier transport properties of organic semiconductors in the frequency domain. In an AS experiment, the electronic device can be used as resistance-capacitor (RC) parallel circuit. We assume that, holes can be injected from the anode and transport through charge transporting network provided by the electron donors, and the cathode is electron-blocking, after applying a direct-current (dc) voltage. Superimpose a small alternating-current (ac) voltage on the dc voltage, resulting in the frequency dependent complex admittance $Y(\omega) = G + i\omega C$. where G represents the conductance, i is the imaginary number and *C* is the capacitance. For $V_{dc}=0$ the capacitance is independent of frequency and equals to geometric capacitance C_{geo}. Plotting the corresponding negative differential susceptance, $-\Delta B$, from the frequency dependent capacitance plot is required to extract the carrier mobility,

$$-\Delta B = -2\pi f\left(C - C_{geo}\right)$$

(S1)

In brief, the maximum of $-\Delta B$ can be obtained at $f=f_r$, in the $-\Delta B$ vs f plot. And the relationship between f_r, and the average carrier transit time τ can be expressed as

$$\tau = \frac{0.56}{f_r} = 0.56\tau_r$$

(S2)

Thus, the carrier mobility (μ) at certain applied voltage (V) can be obtained.

$$\mu = \frac{f_r d^2}{0.56V}$$

(S3)

Instruments and Characterizations

For the tapping atomic force microscopy (AFM) measurements, a Nano Scope IIIA instrument was performed to obtain the height and phase images. Femtosecond transient absorption (TA) spectroscopy measurements were conducted via an Ultrafast Helios pump-probe system combined with a regenerative amplified laser system from Coherent. The time-

resolved terahertz spectroscopy (TRTS) characterization is mainly based on a regeneratively amplified Ti:sapphire laser system. A TOPAS optical parametric amplifier provides the pump pulse and the THz pulse is generated by the optical rectification of 800 nm pulse in a ZnTe crystal. 2D time-resolved fluorescence imaging and steady-state photoluminescence (PL) spectra were recorded by a confocal optical microscope (Nanofinder FLEX2, Tokyo Instruments, Inc.) combined with a time-correlated single-photon counting (TCSPC) module (Becker & Hickl, SPC-150). Temperature-dependent PL spectra were recorded with a spectrometer (PG2000 Pro, Fuxiang Inc.) and the temperature was controlled by a liquid nitrogen chamber (TC280, East Changing, Inc.). The impedance spectroscopy measurement was performed in dark by using an impedance analyzer (E4990A). The grazing-incidence wide-angle X-ray scattering (GIWAXS) characterizations of photoactive layers were performed at BL16B1 beamline at Shanghai Synchrotron Radiation Facility (SSRF), Shanghai, China. The samples for GIWAXS measurements were deposited onto silicon substrates. For GIWAXS characterization, the distances of sample-to-detector and wavelength are 150 mm and 0.154 nm, respectively.

Trap Density Analysis

The trap density can be quantitatively evaluated from capacitance dependent frequency spectra ranging from 1000 Hz to 10 MHz in dark environment. [3–5] The geometric capacitance (C_g) is obtained at 1 MHz from frequency-capacitance spectrum, when the capacitance is independent of the frequency. The ε value is calculated by the equation of

$$\varepsilon = \frac{C_g d}{\varepsilon_0 A}$$

(S4)

where d is the active layer thickness, ε_0 is vacuum dielectric constant and A is the area of device. The deep trap state can be studied at low-frequency response. Here, the additional capacitance at applied voltage (V) close to build-in voltage (V_{bi}) is assumed to be caused by trapped carriers being releasing to the mobility edge. The trapped charge density of accessible trap state can be calculated according to the correlation through Mott–Schottky characterization as follow

$$\frac{1}{C^2} \propto -\frac{2}{N_t \varepsilon q A^2}$$

(S5)

where C is the capacitance N_t is the total trapped charge density, and q is elementary charge.

Calculation of charge carrier density

The density of charge carrier (n) can be determined from capacitance spectroscopy. [6] n can be calculated via the integration of the chemical capacitance using the following equations:

$$n(V_{cor}) = \frac{1}{qAd}\int C_{chem}\, dV_{cor} \qquad \text{(S6)}$$

in which A is the effective area of the OPV cell, d is the thickness of the photoactive layer, V_{cor} is the applied DC voltage corrected by the effect of series resistance ($V_{cor}=V\text{-}JR_S$) where R_S are the series resistance. C_{chem} is calculated by $C_{\text{chem}} = C_{\text{p}}$ (50 KHz) – C_{g} , where C_{g} is the geometric capacitance of the film measured at a reverse bias of -3 V and in the dark, C_P is the capture coefficient for holes. The capacitance-frequency spectra of the three OPV cells measured with bias ranging from -2 V to 2V under dark were summarized in Figure S2.

The tight-binding model and nonadiabatic evolution method

Herein, a detailed description is presented for the three models, including the block copolymer, A-to-A type J-aggregation and A-to-D type J-aggregation (see Figure 1b). For any given model, it is constructed of four repeat units (m=4). Every repeat unit contains one electron-push central fused ring group and two electron-pull terminal groups, forming a linear conjugated backbone with the well-known A-D-A type electronic structure. The sites of the four repeat units are uniformly numbered as n=1→N for the first repeat unit, n=N+1→2N for the second repeat unit, n=2N+1→3N for the third repeat unit, and n=3N+1→4N for the fourth repeat unit, where N=36 indicates the total site number of a repeat unit. The total Hamiltonian of the block copolymer consists of the repeat unit part (H_{repeat}) and the covalent bond linking between two neighboring repeat units (H_{neighbor}). H_{repeat} is written as

$$H_{\text{repeat}} = \sum_j H_{j,\text{repeat}}. \qquad \text{(S7)}$$

$H_{j,\text{repeat}}$ represents the Hamiltonian for the j-th repeat unit, described as

$$\begin{aligned} H_{j,\text{repeat}} = {} & t_{j,n,n+1}(C^+_{j,n+1,s}C_{j,n,s} + C^+_{j,n,s}C_{j,n+1,s}) \\ & + t_3\sum_{l=1}^{4}(C^+_{j,(j-1)N+4l+7,s}C_{j,(j-1)N+4l+10,s} + C^+_{j,(j-1)N+4l+10,s}C_{j,(j-1)N+4l+7,s}) \\ & + t_3\sum_{l=1}^{3}(C^+_{j,(j-1)N+4l+9,s}C_{j,(j-1)N+4l+12,s} + C^+_{j,(j-1)N+4l+12,s}C_{j,(j-1)N+4l+9,s}) \\ & + \Delta_{\text{on}}\sum_{l=1}^{4}(C^+_{j,(j-1)N+4l+7,s}C_{j,(j-1)N+4l+7,s} + C^+_{j,(j-1)N+4l+10,s}C_{j,(j-1)N+4l+10,s}) \\ & + \Delta_{\text{on}}\sum_{l=1}^{3}(C^+_{j,(j-1)N+4l+9,s}C_{j,(j-1)N+4l+9,s} + C^+_{j,(j-1)N+4l+12,s}C_{j,(j-1)N+4l+12,s}) \\ & - \Delta'_{\text{on}}\sum_{n'}C^+_{j,n',s}C_{j,n',s} + \frac{K}{2}\sum_n(u_{j,n+1} - u_{j,n}) + \frac{M}{2}\sum_n \dot{u}^2_{j,n}. \end{aligned}$$

(S8)

The first term represents the electron hopping between the nearest-neighbor sites, and the transfer integral $t_{j,n,n+1}$ between sites n and n+1 is written as

$$t_{j,n,n+1}=\begin{cases} t_0-\alpha(u_{j,n+1}-u_{j,n})-t_1\cos(n\pi/2), & 1+(j-1)N\le n\le 7+(j-1)N, \\ & 29+(j-1)N\le n\le 35+(j-1)N; \\ t_0-\alpha(u_{j,n+1}-u_{j,n}), & 8+(j-1)N\le n\le 10+(j-1)N, \\ & 26+(j-1)N\le n\le 28+(j-1)N; \\ t_0-\alpha(u_{j,n+1}-u_{j,n})-(-1)^n t_2, & 11+(j-1)N\le n\le 25+(j-1)N. \end{cases} \quad \text{(S9)}$$

For any repeat unit, $C^{+}_{j,n,s}(C_{j,n,s})$ denotes the creation (annihilation) operator of an electron with spin s (s=↑, ↓) at site n, $u_{j,n}$ the displacement of site n. α represents the e-l interaction constant. t_0 represents the nearest-neighbor hopping integral for a uniform molecular bond structure. t_1 and t_2 are the symmetry-breaking parameters, separately introduced to describe the lattice feature of terminal groups (t_1) and central group (t_2) of repeat unit. To further consider the effect of heteroatoms X (e.g., S and N atoms) on the central group, we introduce t_3 to describe the electron hopping between the neighboring sites of a heteroatom (see the second and third terms in Eq. (S8)). Most notably, we introduce the on-site energy Δ_{on} in Eq. (S8) to describe the heteroatom contribution to the electron-push ability of the central group, and the on-site energy Δ'_{on} in Eq. (S8) to describe the fluorinated or chlorinated contribution to the electron-pull ability of the terminal groups. In this way, the total electron push-pull ability of a repeat unit can be tuned by the on-site energy difference $\Delta_{\text{on}}+\Delta'_{\text{on}}$ (Δ_{on}=0.6 eV, Δ'_{on}=0 for simplicity). n' represents the site index of the terminal groups of a repeat unit. The last two terms are the lattice part, describing the elastic potential energy and the kinetic energy. K represents the elastic constant and M the mass of a site. H_{neighbor} described as

$$\begin{aligned} H_{\text{neighbor}} &= H_{1-2}+H_{2-3}+H_{3-4} \\ &= -t_{\text{b}}(C^{+}_{1,N,s}C_{2,N+1,s}+C^{+}_{2,N+1,s}C_{1,N,s}) \\ &\quad -t_{\text{b}}(C^{+}_{2,2N,s}C_{3,2N+1,s}+C^{+}_{3,2N+1,s}C_{2,2N,s}) \\ &\quad -t_{\text{b}}(C^{+}_{3,3N,s}C_{4,3N+1,s}+C^{+}_{4,3N+1,s}C_{3,3N,s}). \end{aligned} \quad \text{(S10)}$$

t_{b} shows the hopping integral of the covalent bond linking fragments.

For the A-to-A type J-aggregation and A-to-D type J-aggregation, the total Hamiltonian consists of the repeat unit part (H_{repeat}) with the form of Eq. (S7) and inter-molecular part (H_{inter}). H_{inter} is described as

$$H_{\text{inter}}=H_{1/2}+H_{2/3}+H_{3/4}. \tag{S11}$$

For the A-to-A type J-aggregation

$$\begin{aligned} H_{1/2} &= -t_{\perp}\sum_{n'',s}(C^{+}_{1,n'',s}C_{2,n''+8,s}+C^{+}_{2,n''+8,s}C_{1,n'',s}), \\ H_{2/3} &= -t_{\perp}\sum_{n'',s}(C^{+}_{2,n''+36,s}C_{3,n''+44,s}+C^{+}_{3,n''+44,s}C_{2,n''+36,s}), \\ H_{3/4} &= -t_{\perp}\sum_{n'',s}(C^{+}_{3,n''+72,s}C_{4,n''+80,s}+C^{+}_{4,n''+80,s}C_{3,n''+72,s}). \end{aligned} \tag{S12}$$

For the A-to-D type J-aggregation

$$\begin{aligned} H_{1/2} &= -t_{\perp}\sum_{n'',s}(C^{+}_{1,n'',s}C_{2,n''+18,s}+C^{+}_{2,n''+18,s}C_{1,n'',s}), \\ H_{2/3} &= -t_{\perp}\sum_{n'',s}(C^{+}_{2,n''+36,s}C_{3,n''+54,s}+C^{+}_{3,n''+54,s}C_{2,n''+36,s}), \\ H_{3/4} &= -t_{\perp}\sum_{n'',s}(C^{+}_{3,n''+72,s}C_{4,n''+90,s}+C^{+}_{4,n''+90,s}C_{3,n''+72,s}). \end{aligned} \tag{S13}$$

$t_{\perp}=\frac{t_0}{10}exp[1\text{-}d/5]$ indicates the inter-molecular electron hopping integral between the vertical-neighbor sites of two non-fullerene molecules, determined by the inter-molecular distance d (d=4 Å). $\sum n''$ means the summation only for the sites over the coupling region between molecules.

Now, let us clarify how we get the initial state before dynamical simulations. Placing an electron to the lowest unoccupied molecular orbital level (LUMO), we can get a negative polaron in the block copolymer by iteratively solving the static electronic eigenequation (Eq. (S14)) and the lattice balance equation (Eq. (S15)).

$$\begin{aligned} & t_3\phi_{v,s}(i+3)\left[\delta(\frac{i-7}{4},\text{int})+\delta(\frac{i-9}{4},\text{int})\right] \\ & +t_3\phi_{v,s}(i-3)\left[\delta(\frac{i-10}{4},\text{int})+\delta(\frac{i-12}{4},\text{int})\right] \\ & +\Delta_{\text{on}}\phi_{v,s}(i)-\Delta'_{\text{on}}\phi_{v,s}(n')-t_{n,n+1}\phi_{v,s}(n+1)-t_{n,n-1}\phi_{v,s}(n-1)=\varepsilon_v\phi_{v,s}(n). \end{aligned} \tag{S14}$$

On the other hand, by minimizing the total energy of the molecule, we can obtain the lattice balance equation

$$u_{n+1}-u_n=-\frac{2\alpha}{K}\left(\rho_{n,n+1}-\frac{1}{(mN-1)}\sum_{n=1}^{mN-1}\rho_{n,n+1}\right). \tag{S15}$$

mN is the total site number of a block copolymer molecule. Similarly, by iteratively solving the static electronic eigenequation (Eq. (S16)) and the lattice balance equation (Eq. (S17)), we can get negative polarons in A-to-A type J-aggregation and A-to-D type J-aggregation modes, respectively.

$$\begin{aligned} & t_3\phi_{j,v,s}(i+3)\left[\delta(\frac{i-7}{4},\mathrm{int})+\delta(\frac{i-9}{4},\mathrm{int})\right] \\ & +t_3\phi_{j,v,s}(i-3)\left[\delta(\frac{i-10}{4},\mathrm{int})+\delta(\frac{i-12}{4},\mathrm{int})\right] \\ & +\Delta_{\mathrm{on}}\phi_{j,v,s}(i)-\Delta_{\mathrm{on}}^{'}\phi_{j,v,s}(n')-t_{\perp}\phi_{j',v,s}(n) \\ & -t_{j,n,n+1}\phi_{j,v,s}(n+1)-t_{j,n,n-1}\phi_{j,v,s}(n-1)=\varepsilon_v\phi_{j,v,s}(n). \end{aligned} \quad \text{(S16)}$$

$$u_{j,n+1}-u_{j,n}=-\frac{2\alpha}{K}\left(\rho_{j,n,n+1}-\frac{1}{(N-1)}\sum_{n=1}^{N-1}\rho_{j,n,n+1}\right). \quad \text{(S17)}$$

i represents the site index of central groups. $\delta(x,\mathrm{int})=1$, if x=int; and $\delta(x,\mathrm{int})=0$, if $x\neq$int, where "int" means an integer. j' represents the molecular chain coupled with j. mN is the total site number of a block copolymer molecule. Based on the initial state of the negative polaron at the left lattice point of the molecular chain in the model, we apply an electric field $E(t)$ along the molecular chain to drive the polaron, and the $E(t)$ chosen as a semi-Gaussian form centered at time t_c=30 fs with a width t_w=15 fs,

$$E(t)=\begin{cases} E_0\exp\left[-(t-t_c)^2/t_w^2\right], & t\le t_c, \\ E_0, & t>t_c. \end{cases} \quad \text{(S18)}$$

E_0 refers to the electric field strength. Thus, contribution of the electric field to the block copolymer model can be described as

$$H_E=E(t)\sum_{n,s}e(na+u_n)(C_{n,s}^{+}C_{n,s}-\frac{1}{2}). \quad \text{(S19)}$$

For the A-to-A type J-aggregation model can be described as

$$H_E=\begin{cases} E(t)\sum_{n,s}e(na+u_{j,n})(C_{j,n,s}^{+}C_{j,n,s}-\frac{1}{2}), & 1\le n\le N; \\ E(t)\sum_{n,s}e\left[(n-8)a+u_{j,n}\right](C_{j,n,s}^{+}C_{j,n,s}-\frac{1}{2}), & N+1\le n\le 2N; \\ E(t)\sum_{n,s}e\left[(n-16)a+u_{j,n}\right](C_{j,n,s}^{+}C_{j,n,s}-\frac{1}{2}), & 2N+1\le n\le 3N; \\ E(t)\sum_{n,s}e\left[(n-24)a+u_{j,n}\right](C_{j,n,s}^{+}C_{j,n,s}-\frac{1}{2}), & 3N+1\le n\le 4N. \end{cases} \quad \text{(S20)}$$

For the A-to-D type J-aggregation model can be described as

$$H_E = \begin{cases} E(t)\sum_{n,s} e(na+u_{j,n})(C^{+}_{j,n,s}C_{j,n,s}-\frac{1}{2}), & 1\le n\le N; \\ E(t)\sum_{n,s} e\left[(n-18)a+u_{j,n}\right](C^{+}_{j,n,s}C_{j,n,s}-\frac{1}{2}), & N+1\le n\le 2N; \\ E(t)\sum_{n,s} e\left[(n-36)a+u_{j,n}\right](C^{+}_{j,n,s}C_{j,n,s}-\frac{1}{2}), & 2N+1\le n\le 3N; \\ E(t)\sum_{n,s} e\left[(n-54)a+u_{j,n}\right](C^{+}_{j,n,s}C_{j,n,s}-\frac{1}{2}), & 3N+1\le n\le 4N. \end{cases} \quad \text{(S21)}$$

where *e* indicates the electronic charge and *a* the lattice constant (*a*=1.22 Å). When the *E*(*t*) is turned on, the system will experience an evolution. By using a nonadiabatic evolution method, we can separately obtain the temporal evolution of the electronic state $\Psi_{\nu,s}(n,t)$ and the lattice displacement $u_n(t)$. For the block copolymer, the evolution of an electronic state $\Psi_{\nu,s}(n,t)$ depends on the time dependent Schrödinger equation:

$$\begin{aligned} i\hbar\frac{\partial\Psi_{\nu,s}(n,t)}{\partial t} &= -t_{n,n+1}\Psi_{\nu,s}(n+1,t)-t_{n-1,n}\Psi_{\nu,s}(n-1,t) \\ &+t_3\{\Psi_{\nu,s}(i+3,t)[\delta\left(\frac{i-7}{4},\text{int}\right)+\delta\left(\frac{i-9}{4},\text{int}\right)] \\ &+\Psi_{\nu,s}(i-3,t)[\delta\left(\frac{i-10}{4},\text{int}\right)+\delta\left(\frac{i-12}{4},\text{int}\right)]\} \\ &+\Delta_{\text{on}}\Psi_{\nu,s}(i,t)-\Delta'_{\text{on}}\Psi_{\nu,s}(n',t) \\ &+eE(t)(na+u_n)\Psi_{\nu,s}(n,t). \end{aligned} \quad \text{(S22)}$$

$\Psi_{\nu,s}(n,t)=\langle n|\Psi_{\nu,s}(t)\rangle$ is projection of electronic state $|\Psi_{\nu,s}(t)\rangle$ on the Wannier state of site *n*. For the lattice part, the nuclear motion is classically described by the Newtonian equation of motion:

$$\begin{aligned} M\ddot{u}_n &= -K(2u_n-u_{n+1}-u_{n-1})+2\alpha\left[\rho_{n,n+1}(t)-\rho_{n-1,n}(t)\right] \\ &+eE(t)\left[\rho_{n,n}(t)-1\right]-\lambda M\dot{u}_n. \end{aligned}$$

(S23)

The density matrix $\rho_{n,m}$ is defined as:

$$\rho_{n,m}(t)=\sum_{\nu,s}\Psi^{*}_{\nu,s}(n,t)\,g_{\nu,s}\sum_{\nu,s}\Psi_{\nu,s}(m,t). \quad \text{(S24)}$$

Here, $g_{\nu,s}$ is a time-independent distribution function and determined by the initial occupation of the electronic state $|\Psi_{\nu,s}(t)\rangle$. Eq. (S23) and (S24) can be numerically solved by

the Runge-Kutta method of order eight with step-size control. Similarly, for the A-to-A type J-aggregation and A-to-D type J-aggregation mode, the evolution of an electronic state $\Psi_{j,\nu,s}(n,t)$ depends on the time dependent Schrödinger equation:

$$\begin{aligned} i\hbar\frac{\partial\Psi_{j,\nu,s}(n,t)}{\partial t} &= -t_{j,n,n+1}\Psi_{j,\nu,s}(n+1,t)-t_{n-1,n}\Psi_{j,\nu,s}(n-1,t) \\ &+t_3\{\Psi_{j,\nu,s}(i+3,t)[\delta\left(\frac{i-7}{4},\text{int}\right)+\delta\left(\frac{i-9}{4},\text{int}\right)] \\ &+\Psi_{j,\nu,s}(i-3,t)[\delta\left(\frac{i-10}{4},\text{int}\right)+\delta\left(\frac{i-12}{4},\text{int}\right)]\} \\ &+\Delta_{\text{on}}\Psi_{j,\nu,s}(i,t)-\Delta'_{\text{on}}\Psi_{j,\nu,s}(n',t) \\ &+eE(t)x_n\Psi_{j,\nu,s}(n,t)-t_{\perp}\Psi_{j',\nu,s}(n,t). \end{aligned} \quad \text{(S25)}$$

$\Psi_{j,\nu,s}(n,t)=\langle n|\Psi_{j,\nu,s}(t)\rangle$ is projection of electronic state $|\Psi_{j,\nu,s}(t)\rangle$ on the Wannier state of site *n*. x_n is the site of the negative polaron. For the lattice part, the nuclear motion is classically described by the Newtonian equation of motion:

$$\begin{aligned} M\ddot{u}_{j,n} &= -K(2u_{j,n}-u_{j,n+1}-u_{j,n-1})+2\alpha\left[\rho_{j,n,n+1}(t)-\rho_{j,n-1,n}(t)\right] \\ &+eE(t)\left[\rho_{j,n,n}(t)-1\right]-\lambda M\dot{u}_{j,n}. \end{aligned} \quad \text{(S26)}$$

The density matrix $\rho_{j,n,m}$ is defined as:

$$\rho_{j,n,m}(t)=\sum_{\nu,s}\Psi^*_{j,\nu,s}(n,t)g_{\nu,s}\sum_{\nu,s}\Psi_{j,\nu,s}(m,t). \quad \text{(S27)}$$

Eq. (S26) and (S27) can be numerically solved by the Runge-Kutta method of order eight with step-size control. In addition, a damping term is introduced in Eq. (S23) and (S26) to describe the energy dissipation into the surrounding medium by a tuning a parameter λ=0.05 fs^{-1} . In all simulations, the values of model parameters are set as t_0=2.5 eV, t_1=t_2=0.05 eV, t_3= 0.1 eV, t_b= 1.5 eV, M=1349 eV · fs^2/Å^2, α=4.1 eV/Å and K=25 eV/Å^2 without specific clarifications.

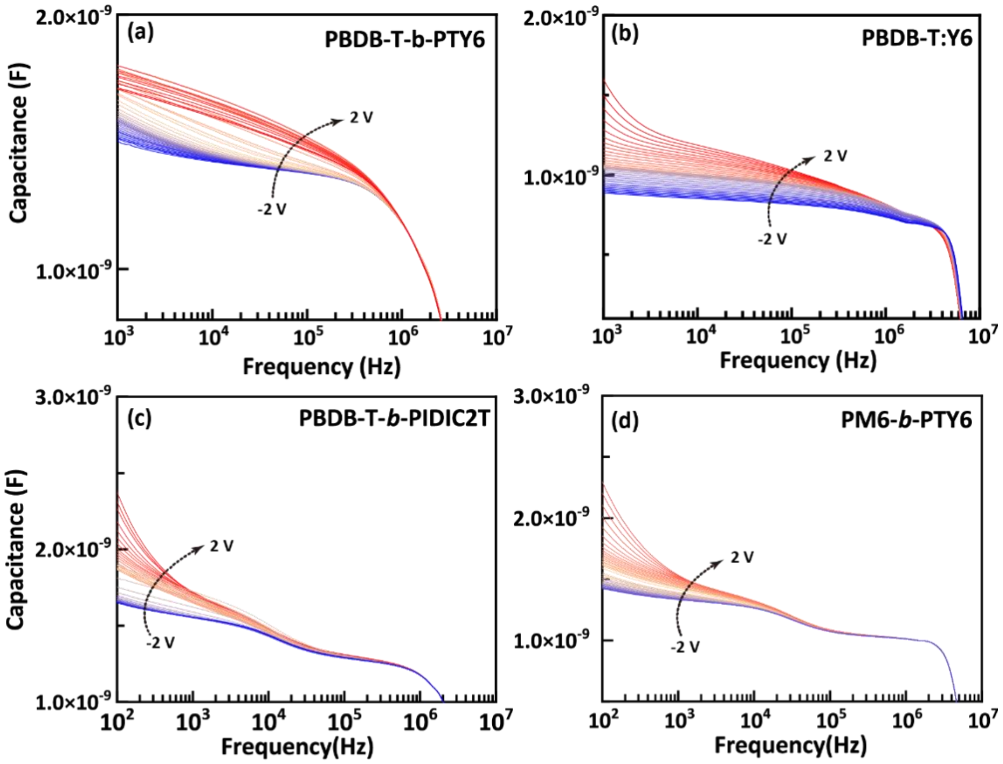

Fig. S1. Bias voltage dependent capacitance-frequency spectra of the BHJ-type and single-component OSCs under dark conditions.

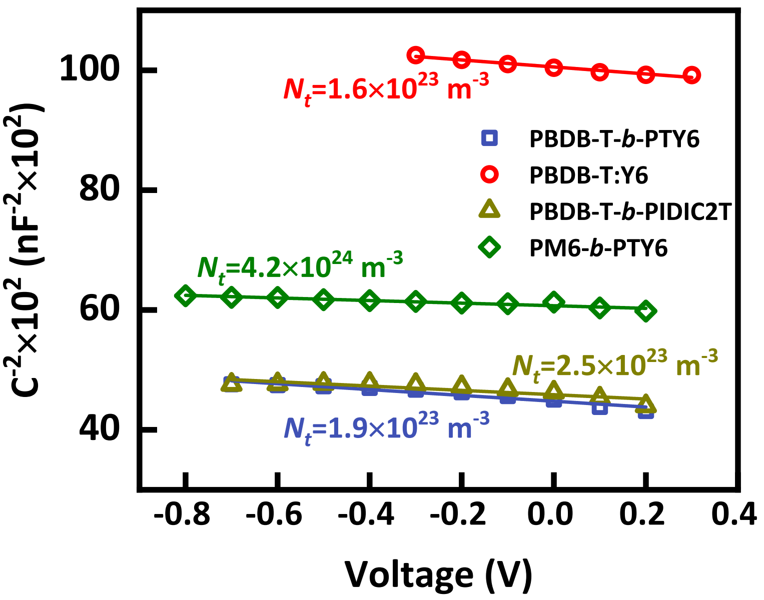

Fig. S2. The C^{-2} versus V plots were extracted at 10k Hz for different types of BHJ-type and single-component OSCs.

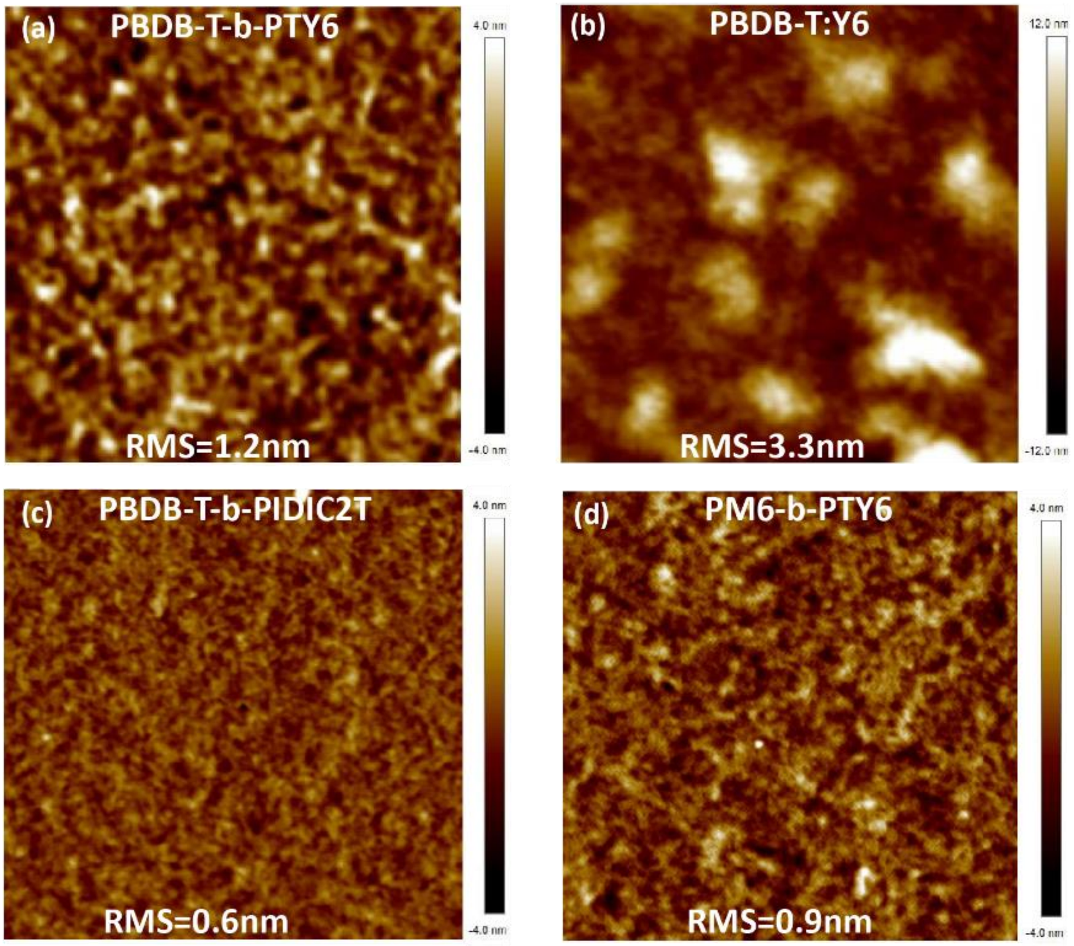

Fig. S3. Atomic force microscopy (AFM) height images of block-polymer-based films and BHJ-type film.

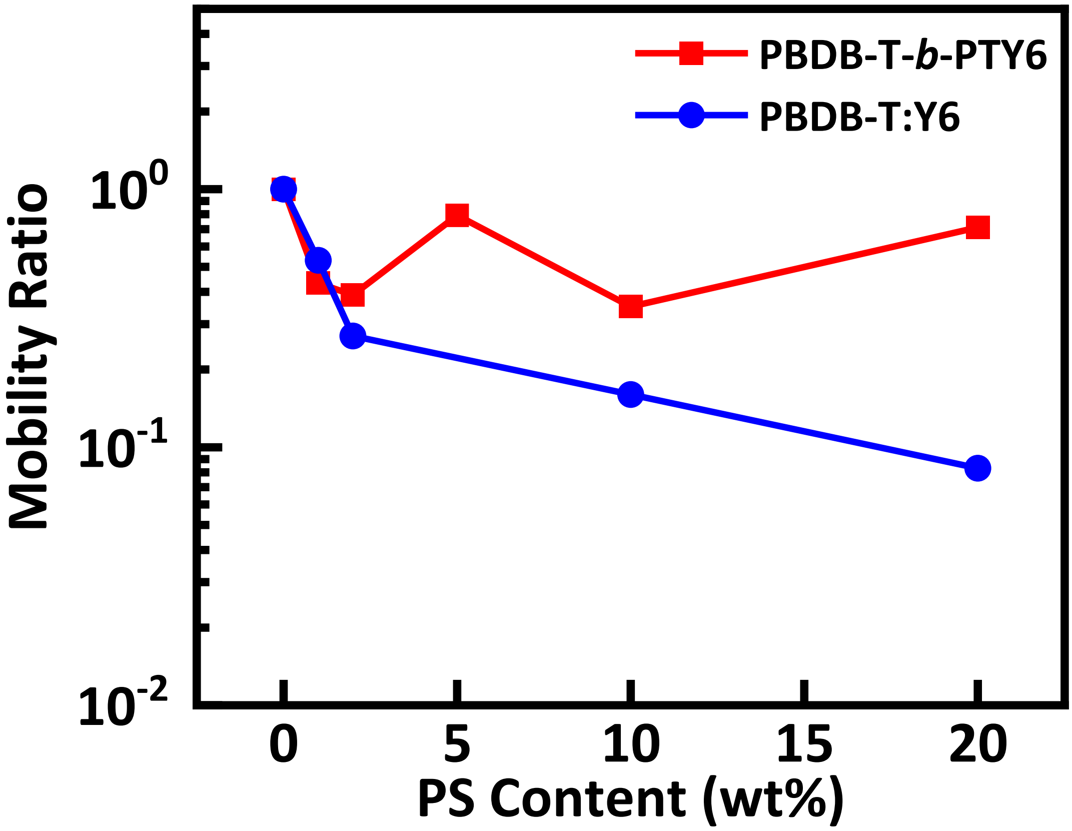

Fig. S4. The hole mobility change ratio of single-component material PBDB-T-*b*-PTY6 and the counterpart PBDB-T:Y6 BHJs with different PS contents.

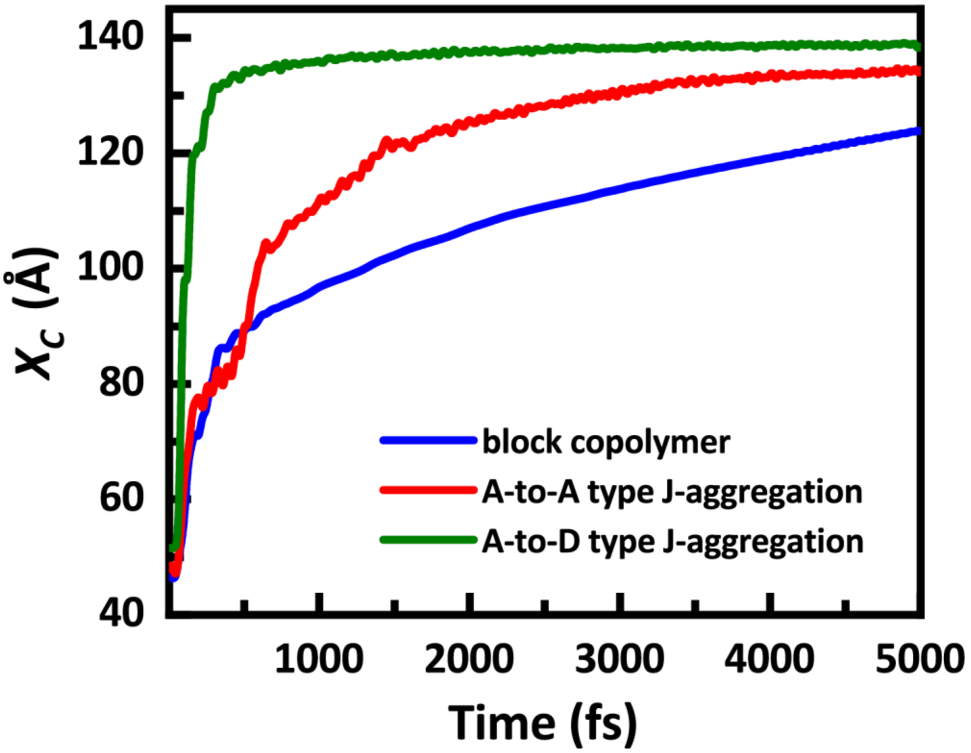

Fig. S5. The evolution of the charge center position ($x_c=\frac{\sum_n a(n-1)q_n}{\sum_n q_n}$) of the negative polaron in the block copolymer, A-to-A type J-aggregation and A-to-D type J-aggregation structural models under an applied electric field of E_0 = 4×10^5 V/cm.

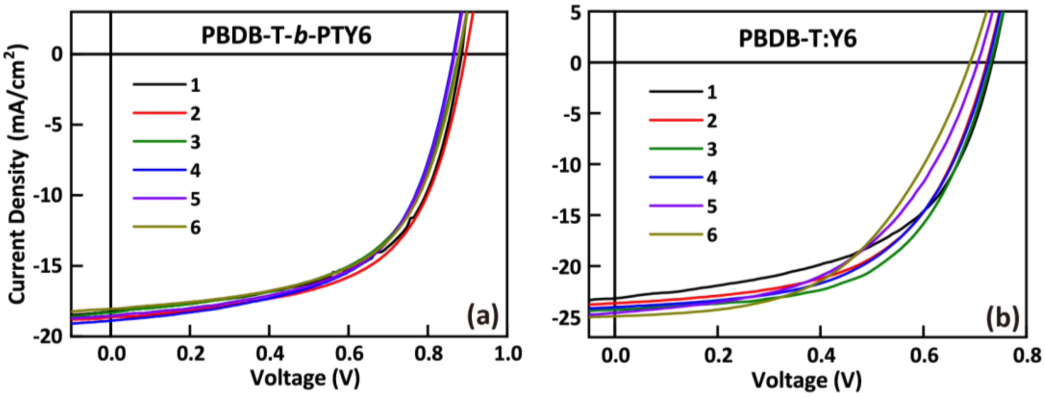

Fig. S6. J-V characteristics of different OPV devices based on (a) single-component PBDB-T-*b*-PTY6 and (b) PBDB-T:Y6 blend solar cells under 1-Sun illumination.

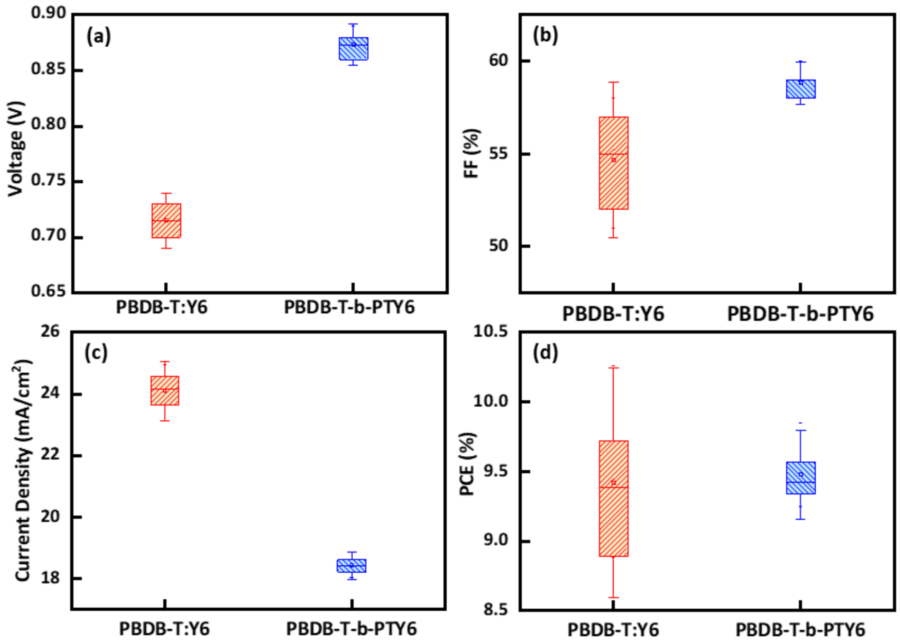

Fig. S7. The parameter distribution for (a) open circuit voltage (V_{OC}), (b) fill factor (*FF*), (c) short circuit current density (J_{SC}) and (d) power conversion efficiency (PCE) of the different organic solar cells based on PBDB-T:Y6 BHJ blended and PBDB-T-*b*-PTY6 block-polymer-based films under the AM 1.5G illumination.

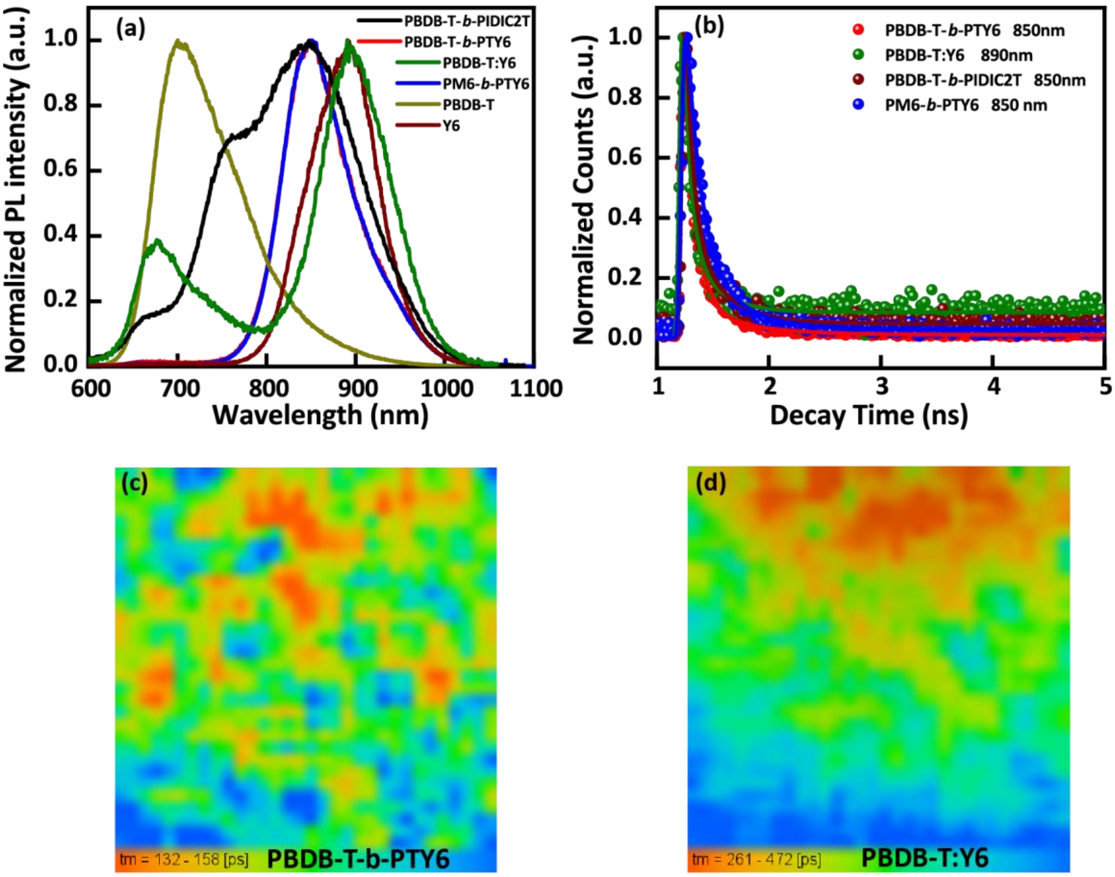

Fig. S8. (a) Normalized PL spectra of PBDB-T, Y6, BHJ-type and single-component films; (b) Normalized TRPL decay traces of BHJ-type and single-component films. 2D time-resolved fluorescence images of (c) PBDB-T-*b*-PTY6 and (d) PBDB-T:Y6 BHJ devices under 400 nm excitation.

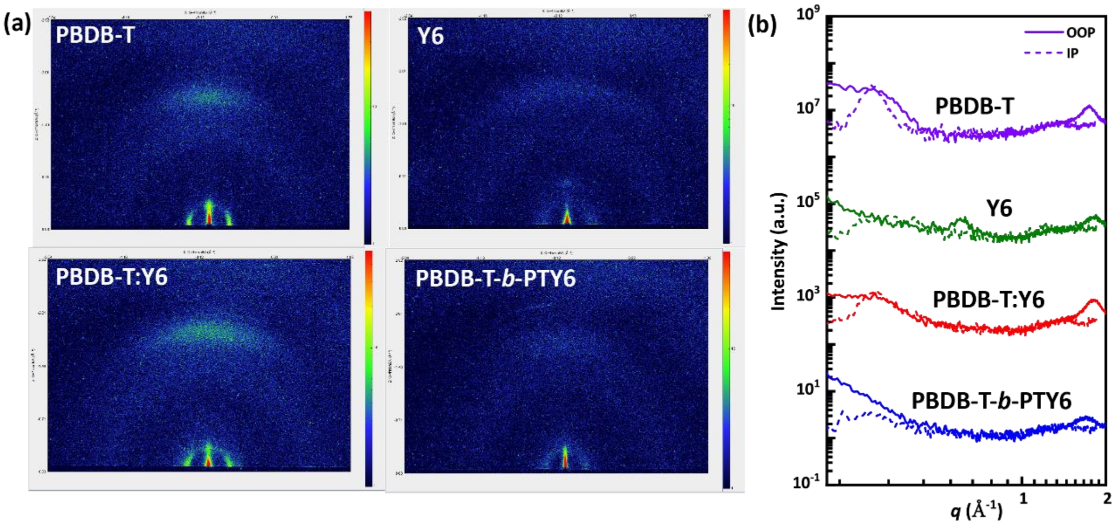

Fig. S9. (a) 2D GIWAXS diffraction patterns of neat PBDB-T, neat PTY6, and PBDB-T:Y6 BHJ blend and PBDB-T-*b*-PTY6 block-polymer-based films on Si substrate. (b) The one-dimensional scattering profiles of the corresponding films with respect to the OOP and IP directions.

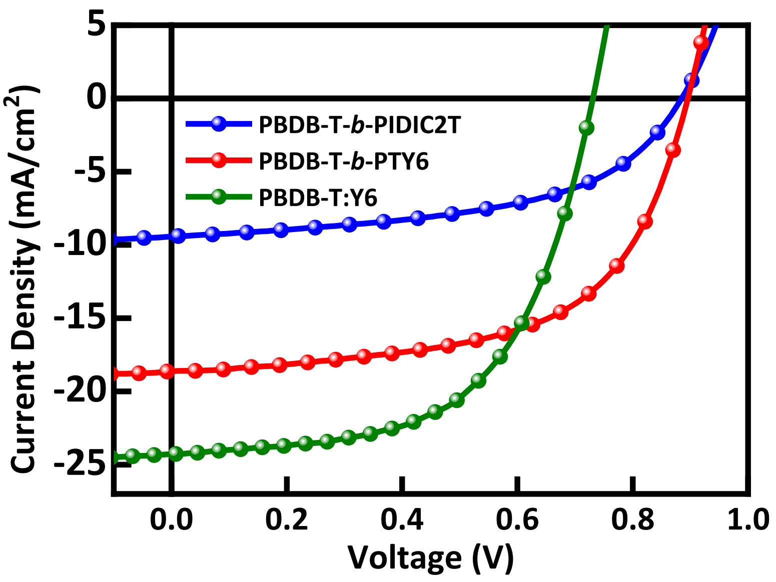

Fig. S10. J-V characteristics of OPV devices based on single component PBDB-T-*b*-PIDIC2T, PBDB-T-*b*-PTY6 and PBDB-T:Y6 blend solar cells under 1-Sun illumination.

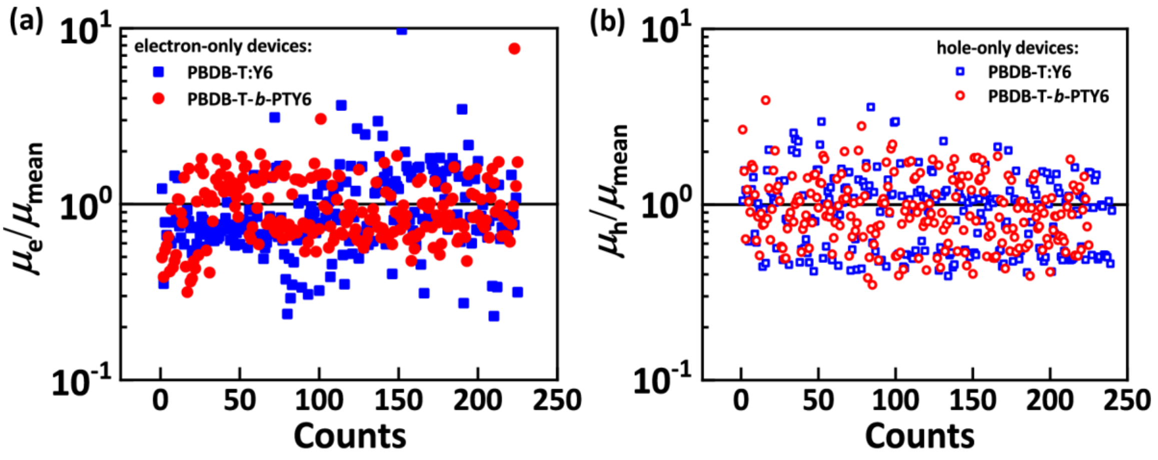

Fig. S11. The ratio of carrier mobility of at least 200 single-carrier devices based on single component PBDB-T-*b*-PTY6 and PBDB-T:Y6 blend solar cells to their overall mean mobility for (a) electron-only and (b) hole-only devices.

Table S1. The value of electron energetic disorder σ_e of different single component-type and BHJ-type electron-only and hole-only devices.

**Devices**	**Energetic disorder σ_e (*meV*)**
PBDB-T-b-PIDIC2T	62
PM6-b-PTY6	72
PBDB-T-b-PTY6	60
PBDB-T:Y6	54

Table S2. Details of the photovoltaic parameters of single component-type OSCs.

**Single component-type OSCs**	**V_{OC} (V)**	**J_{SC} (mA/cm^2)**	**FF (%)**	**PCE (%)**
PBDB-T-b-PIDIC2T	0.88	9.39	52.74	4.36
PBDB-T-b-PTY6	0.89	18.62	59.16	9.85
PM6-b-PTY6	0.93	8.45	41.79	3.27

Table S3. Details of the photovoltaic parameters of BHJ-type OSCs.

BHJ-type OSCs	V_{OC} (V)	J_{SC} (mA/cm^2)	FF (%)	PCE (%)
PBDB-T:Y6	0.72	24.10	54.66	9.42

Table S4. Details of the exciton lifetime of single component-type and BHJ-type devices.

**Devices**	**τ_1 (ps)**	**A_1 (%)**	**τ_2 (ps)**	**A_2 (%)**	**τ_m (ps)**
PBDB-T-b-PTY6	85.0	90.3	447.4	9.7	120.12
PBDB-T-b-PIDIC2T	88.3	84.06	356.5	15.94	131.09
PM6-b-PTY6	158.7	90.93	611.1	9.07	199.68
PBDB-T:Y6	87.5	95.02	620.7	4.98	114.08

Table S5. The important parameters and the charge carrier mobilities of single- component-type and BHJ-type electron-only devices through SCLC and trap-SCLC.

Device	PBDB-T:Y6	PBDB-T-*b*-PTY6	PBDB-T-b-PIDIC2T	PM6-*b*-PTY6
Slope	2.0	3.8	4.1	3.6
N_t ($10^{23}m^{-3}$)	1.5	1.9	2.5	4.2
n_0 ($10^{21}m^{-3}$)	/	16	2.7	4.3
ϑ	/	0.08	0.01	0.01
$\mu_{e,SCLC}$ (10^{-4} $cm^2V^{-1}s^{-1}$)	6.0	0.069	0.00028	0.051
$\mu_{e,Trap\text{-}SCLC}$ (10^{-4} $cm^2V^{-1}s^{-1}$)	/	0.87	0.028	5.1

Table S6. Morphology parameters of out-of-line analysis obtained from GIWAXS curves.

Devices	$q_{z(010)}$ [nm-1]	*d-spacing* [nm]
PBDB	17.2	0.365
Y6	17.5	0.359
PBDB-T:Y6	17.7	0.355
PBDB-T-b-PTY6	16.9	0.371

References

[1] S. Li, X. Yuan, Q. Zhang, B. Li, Y. Li, J. Sun, Y. Feng, X. Zhang, Z. Wu, H. Wei, M. Wang, Y. Hu, Y. Zhang, H. Y. Woo, J. Yuan, W. Ma, *Adv. Mater.* **2021**, *33*, 2101295.
[2] B. Li, Q. Zhang, S. Li, X. Yang, F. Yang, Y. Kong, Y. Li, Z. Wu, W. Zhang, Q. Zhao, Y. Zhang, H. Young Woo, J. Yuan, W. Ma, *Chem. Eng. J.* **2022**, *438*, 135543.
[3] S. Khelifi, K. Decock, J. Lauwaert, H. Vrielinck, D. Spoltore, F. Piersimoni, J. Manca, A. Belghachi, M. Burgelman, *J. Appl. Phys.* **2011**, *110*, 094509.
[4] M. Zhang, L. Zhu, T. Hao, G. Zhou, C. Qiu, Z. Zhao, N. Hartmann, B. Xiao, Y. Zou, W. Feng, H. Zhu, M. Zhang, Y. Zhang, Y. Li, T. P. Russell, F. Liu, *Adv. Mater.* **2021**, *33*, 2007177.
[5] Y. Zhang, G. Cai, Y. Li, Z. Zhang, T. Li, X. Zuo, X. Lu, Y. Lin, *Adv. Mater.* **2021**, *33*, 2008134.
[6] A. Karki, J. Vollbrecht, A. J. Gillett, P. Selter, J. Lee, Z. Peng, N. Schopp, A. L. Dixon, M. Schrock, V. Nádaždy, F. Schauer, H. Ade, B. F. Chmelka, G. C. Bazan, R. H. Friend, T. Nguyen, *Adv. Energy Mater.* **2020**, *10*, 2001203.
